# Treatment of Pelvic Organ Prolapse in a Patient with a Thermal Burn Wound Caused by Hot Stone Therapy, a Traditional Thai Treatment

**DOI:** 10.1155/2017/4925124

**Published:** 2017-12-13

**Authors:** Sasivimol Srisukho, Orawee Chinthakanan

**Affiliations:** ^1^Department of Obstetrics and Gynecology, Faculty of Medicine, Chiang Mai University, Chiang Mai, Thailand; ^2^Female Pelvic Medicine and Reconstructive Surgery Division, Department of Obstetrics & Gynecology, Ramathibodi Hospital, Mahidol University, Bangkok, Thailand

## Abstract

A 72-year-old woman presented with a 1-month history of an incarcerated uterine prolapse along with an infected wound at the anterior uterine wall. She had previously undergone the traditional Thai practice Yue Fai, or “lying by the fire,” as performed by postpartum women. However, her uterus was burned by the extremely high temperature involved in the practice; it subsequently became infected and incarcerated. Pelvic examination revealed stage IV genitourinary prolapse according to the POP-Q classification. An ill-defined ulcer measuring 6.5 × 4.5 cm was present in the anterior wall of the uterus, and a 2.0 cm diameter ulcer was present in the right posterior wall of the uterus. The patient was treated symptomatically with broad-spectrum antibiotics, local estrogen therapy, analgesic and anti-inflammatory agents, and antiseptic dressing of the ulcerated area. After alleviation of all symptoms, the ulcer almost completely healed. She was advised to undergo definitive surgical treatment for the prolapsed uterus.

## 1. Introduction

The prevalence of pelvic organ prolapse (POP) in low- and middle-income countries is 19.7% (range, 3.4%–56.4%) [1]. The symptoms vary in severity. The exposed mass is vulnerable to trauma and infection. Women who experience distressful symptoms of POP usually have an impaired quality of life [2]. Many women do not seek medical advice because they feel shameful. As a result, patients with POP often attempt self-treatment [3]. In this case report, we describe a 72-year-old woman with stage IV POP who sustained a thermal burn and infection of her uterus after undergoing hot stone therapy.

## 2. Case Report

**Figure 1:**
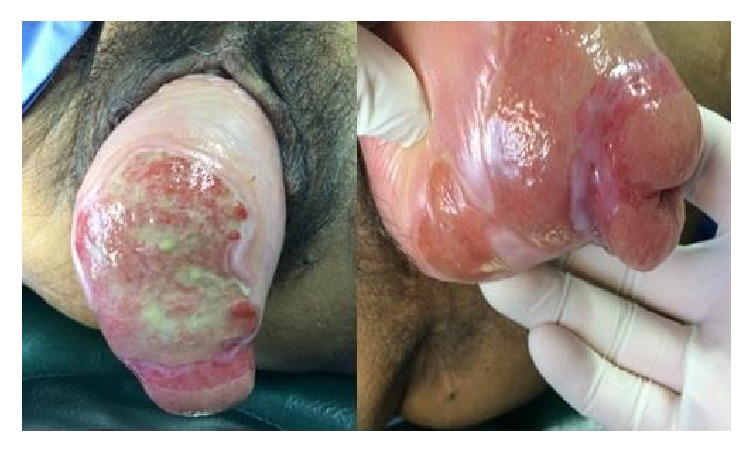
The prolapsed uterus was nonreducible due to the inflamed and edematous vaginal mucosa. An ill-defined ulcer measuring 5 × 6 cm was present in the anterior wall of the uterus with marked edema and ulceration of the surrounding tissue.

**Figure 2:**
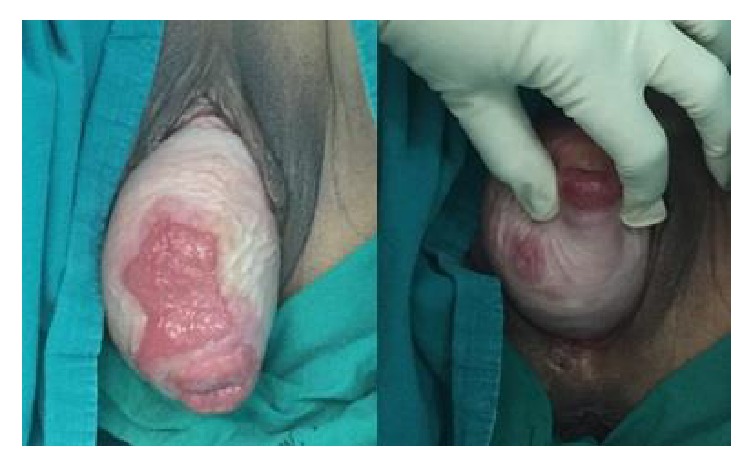
After treatment with broad-spectrum antibiotics, local estrogen therapy, analgesic and anti-inflammatory agents, and antiseptic dressing of the ulcerated area, the ulcer almost completely healed. The patient provided written informed consent for publication of these photographs.

A 72-year-old sexually active para 2 living in Southern Thailand presented at the outpatient department of our clinic with a 1-month history of an incarcerated uterine prolapse along with an infected wound at the anterior wall of the uterus. She had a 3-year history of a reducible prolapsed uterus and had experienced no symptoms of urinary discomfort or difficulty with defecation. During the month before the current presentation, she had attempted to treat herself with hot stone therapy similar to that practiced by some postpartum women. She believed that placing a heated stone on her uterus would return it to the normal position. However, her uterus was burned. It became swollen, incarcerated, and finally infected because of several underlying illnesses including hypertension, dyslipidemia, and especially poorly controlled diabetes mellitus. Pelvic examination revealed a stage IV genitourinary prolapse according to the POP-Q classification. An ill-defined ulcer measuring 6.5 × 4.5 cm was present in the anterior vaginal wall, and a 2.0 cm diameter ulcer was present in the right posterior vaginal wall (Figure 1). The prolapsed uterus was nonreducible due to the inflamed, edematous vaginal mucosa. The patient was treated symptomatically with broad-spectrum antibiotics, local estrogen therapy, analgesic and anti-inflammatory agents, and antiseptic dressing of the ulcerated area. After alleviation of all symptoms, the ulcer almost completely healed (Figure 2). She was advised to undergo definitive surgical treatment for the prolapsed uterus. Six weeks after presentation, the patient underwent a vaginal hysterectomy with McCall culdoplasty and anterior and posterior repairs under spinal anesthesia. Intraoperative cystoscopy showed multiple calculi within the dependent portion of the bladder. The bladder was copiously irrigated. The patient was doing well at her 3-month postoperative visit without recurrence of the prolapse and her postvoid residual urine volume was <50 ml. A follow-up cystoscopic examination revealed no bladder calculi. The pathologic examination of the uterus showed no evidence of malignancy.

## 3. Discussion

**Figure 3:**
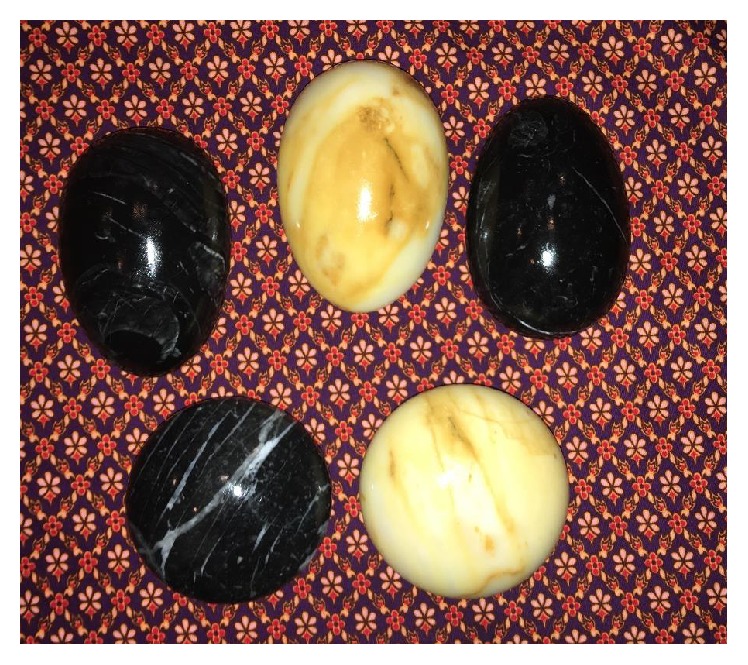
A hot stone therapy which is a part of traditional Thai postpartum practices is believed to promote uterus involution and help to heal perineal tears in postpartum period.

POP is a silent problem due to patient nondisclosure and embarrassment; moreover, some patients have misperceptions of available treatment options [4–6]. Although POP is not life-threatening, it can impose significant burdens with respect to social and physical activity restrictions and a detrimental impact on the patient's psychological well-being and overall quality of life. Some women attribute their delay in seeking treatment to a lack of information and attempt self-treatment. Some women who live in rural areas habitually do not seek care from a health care provider in addition to practicing self-treatment behaviors. Our patient decided to treat her prolapsed uterus using hot stone therapy (Figure 3) because of her postpartum experiences. Traditional Thai postpartum practices include restricting food, taking hot baths, consuming hot drinks, and applying pressure with hot objects. Reheating the body is believed to promote uterus involution and help to heal perineal tears in the postpartum period. This belief was similar to the treatment of POP over the centuries in the past. According to the history of treatment of POP [7], we found that many remedies such as honey, hot oil, wine, and fumigations had been used in prolapsed uteri. In our case, the patient applied this remedy by pressing the hot stone directly onto her prolapsed uterus in an effort to reduce the prolapse. However, the hot stone heated the anterior uterine wall, resulting in a thermal burn wound in this area. Furthermore, her poorly controlled diabetes mellitus prevented healing of the wound, promoting the development of infection, incarceration, and ulcer formation on the posterior wall of the uterus. A literature review revealed a case of a thermal burn of a prolapsed uterus in 1997 as reported by Sagi et al. [8]. In that case, a 72-year-old Bedouin woman with uterine prolapse sustained burns of 70% of her total body surface area from an open fire. Unfortunately, their patient did not survive the burn. The authors considered that the risk of peritonitis and generalized infection is potentially high because the lymphatics of the uterus drain directly into the abdominal cavity; therefore, they recommended that any local and systematic therapy administered must protect against uterine pathogens.

Longstanding untreated POP, especially severe cases, may cause renal complications such as acute renal failure, uremia, and bladder stones. In previous reports of patients with incarcerated procidentia [9, 10], we found that cystolithiasis should be considered after completion of reconstructive surgery. Therefore, in the present case, we performed cystourethroscopy and removed many bladder stones.

In conclusion, patients' lack of knowledge about POP may lead to hesitancy in seeking medical care. The present case emphasizes the importance of health education programs. The local public health unit in each community must take action to provide accurate information to women to prevent complications from self-treatment.
